# Evidence of high-temperature exciton condensation in a two-dimensional semimetal

**DOI:** 10.1038/s41467-023-36667-x

**Published:** 2023-02-22

**Authors:** Qiang Gao, Yang-hao Chan, Yuzhe Wang, Haotian Zhang, Pu Jinxu, Shengtao Cui, Yichen Yang, Zhengtai Liu, Dawei Shen, Zhe Sun, Juan Jiang, Tai C. Chiang, Peng Chen

**Affiliations:** 1 Key Laboratory of Artificial Structures and Quantum Control (Ministry of Education), Shenyang National Laboratory for Materials Science, Shanghai Center for Complex Physics, School of Physics and Astronomy, Shanghai Jiao Tong University, 200240 Shanghai, China; 2 Institute of Atomic and Molecular Sciences, Academia Sinica, Taipei, 10617 Taiwan; 3 Physics Division, National Center for Theoretical Sciences, Taipei, 10617 Taiwan; 4 School of Future Technology, University of Science and Technology of China, 230026 Hefei, Anhui China; 5 National Synchrotron Radiation Laboratory, University of Science and Technology of China, 230026 Hefei, Anhui China; 6 State Key Laboratory of Functional Materials for Informatics, Shanghai Institute of Microsystem and Information Technology (SIMIT), Chinese Academy of Sciences, 200050 Shanghai, China; 7 Department of Physics, University of Illinois at Urbana-Champaign, 1110 West Green Street, Urbana, Illinois 61801-3080 USA; 8 Frederick Seitz Materials Research Laboratory, University of Illinois at Urbana Champaign, 104 South Goodwin Avenue, Urbana, Illinois 61801-2902 USA

**Keywords:** Electronic properties and materials, Surfaces, interfaces and thin films, Two-dimensional materials

## Abstract

Electrons and holes can spontaneously form excitons and condense in a semimetal or semiconductor, as predicted decades ago. This type of Bose condensation can happen at much higher temperatures in comparison with dilute atomic gases. Two-dimensional (2D) materials with reduced Coulomb screening around the Fermi level are promising for realizing such a system. Here we report a change in the band structure accompanied by a phase transition at about 180 K in single-layer ZrTe_2_ based on angle-resolved photoemission spectroscopy (ARPES) measurements. Below the transition temperature, gap opening and development of an ultra-flat band top around the zone center are observed. This gap and the phase transition are rapidly suppressed with extra carrier densities introduced by adding more layers or dopants on the surface. The results suggest the formation of an excitonic insulating ground state in single-layer ZrTe_2_, and the findings are rationalized by first-principles calculations and a self-consistent mean-field theory. Our study provides evidence for exciton condensation in a 2D semimetal and demonstrates strong dimensionality effects on the formation of intrinsic bound electron–hole pairs in solids.

## Abstract (web summary)

Two-dimensional materials are promising platforms for the realization of an excitonic insulator state. Here the authors report evidence for an excitonic insulator in a single-layer ZrTe2 based on ARPES measurements.

## Introduction

Excitons in solids, bound states between an electron and a hole, were predicted to condense under appropriate conditions^1–8^. Because of their small effective masses, such condensation can occur at high temperatures^9,10^, which is of great interest in modern condensed matter research. Although the condensed phases of excitons have been realized in cases involving optical pumping, quantum Hall states in a magnetic field, and electrical generation in atomic double-layer heterostructures^9–14^, very few examples were identified in which the long-range excitonic order is spontaneously formed at low temperatures under equilibrium conditions. Realization of such a system is difficult because it needs a delicate balance among several factors: the size of the band gap, the binding energy of the electron–hole pairs, and the screening strength^5,7^. Promising candidates so far include TmSe_0.45_Te_0.55_ under pressure, Ta_2_NiSe_5_, 1*T*-TiSe_2_, and other proposed candidates^15–29^. It is still under intense debate whether the phase transition in Ta_2_NiSe_5_ is driven by an exciton condensation or the lattice distortion from a change of the structure^22,23,27,28^, and various competing interpretations have been offered for bulk TiSe_2_^18,20^. Thus far, experimental observation of spontaneous exciton condensation remains inconclusive.

**Fig. 1:**
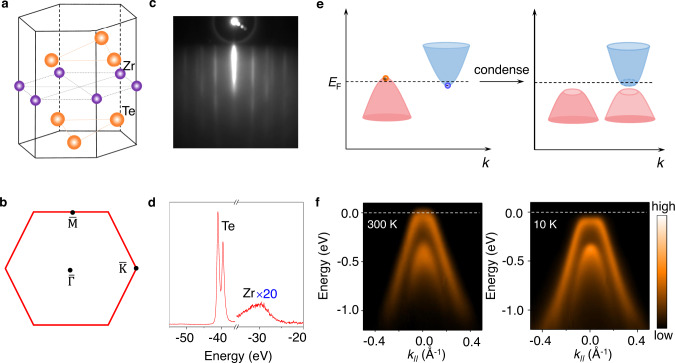
Film structure and electronic band structure of single-layer ZrTe2. **a** Atomic structure of single-layer ZrTe_2_. **b** Corresponding 2D Brillouin zone. **c** A RHEED pattern of single-layer ZrTe_2_ film taken at room temperature. **d** Core-level photoemission spectra taken with 100 eV photons. **e** Schematic diagram for evolution of the band structure and the opening of a gap from a semimetal during the exciton condensation. **f** ARPES maps along $\overline{\Gamma {{{{{\rm{M}}}}}}}$ taken with *s* polarized light for the normal phase at 300 K and the condensed phase at 10 K.

Exciton condensation in materials has been predicted to lead to a new insulating ground state, an excitonic insulator^4–8^. General features related to the phase transition include the mixing of conduction and valence bands and gap opening around the Fermi level (*E*_F_). The band structure is renormalized and exhibits a characteristic flat valence band top (Fig. 1)^29^. These signatures should be readily captured by ARPES. Transition metal dichalcogenides with vanishing indirect band gap or band overlap between the valence and conduction bands are promising candidates for developing 2D excitonic insulators^4–7^. These layered materials can be easily prepared as single layers, and single-layer TiSe_2_ appears to be a natural choice. However, the semimetal-insulator transition and a flat valence band top have not been observed^30^. Another strong candidate is ZrTe_2_; in bulk form, it is a metal/semimetal with a negative band gap of about −0.5 eV^31,32^. The Coulomb interaction between electrons and holes is strongly screened by the many carriers around the metallic Fermi surfaces, and no exciton condensation is expected or observed. This unfavorable condition is, however, completely reversed for the single layer, for which the gap is almost zero, and electronic screening is largely suppressed in the 2D limit^26,33^.

## Results

### Electronic band structure of single-layer ZrTe_2_

Here we study the band structures of single-layer ZrTe_2_ and present experimental evidence of high-temperature exciton condensation. The structure of single-layer ZrTe_2_ (Fig. 1a) in the normal phase consists of a triangular planar net of Zr atoms sandwiched between two Te atomic layers. Sharp reflection high-energy electron diffraction (RHEED) patterns (Fig. 1c) reveal a high-quality and well-ordered single layer of ZrTe_2_ grown by molecular beam epitaxy (MBE) on a bilayer-graphene-terminated SiC substrate. Angle-integrated core-level scans (Fig. 1d) show the characteristic peaks of Zr and Te for the 1*T* phase^31^. Figure 1f shows ARPES maps taken with *s* polarized light along the $\overline{\Gamma {{{{{\rm{M}}}}}}}$ direction. Two hole-like valence bands are seen centered at the $\overline{\Gamma }$ point. A previous study of single-layer ZrTe_2_ grown on InAs (111) shows a similar band structure, but with an upward shift of the Fermi level. The difference suggests a charge transfer from the InAs substrate^32^. For our sample at 300 K, the conduction band edge at the $\overline{{{{{{\rm{M}}}}}}}$ point reaches just around the Fermi level. With the valence band edge just slightly above the Fermi level, the system is metallic. The indirect band overlap around the Fermi level is only about −0.06 eV (Supplementary Fig. 2), much smaller than the bulk case. A small band overlap implicates a small carrier density around the Fermi level, which is favorable for the formation of excitons.

**Fig. 2:**
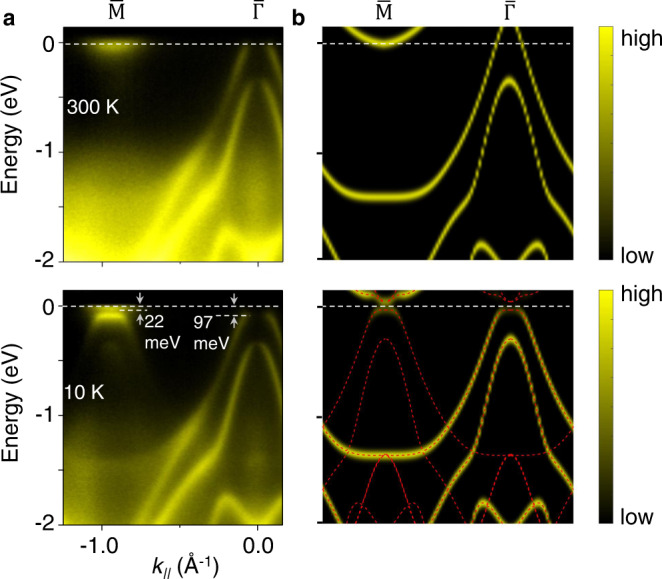
Condensation-induced band gap opening and calculated spectra function with exciton order. **a** ARPES spectra taken with *p* polarized light along $\overline{\Gamma {{{{{\rm{M}}}}}}}$ direction at 300 K and 10 K. The data at 10 K shows the gap opening and folded bands around the $\overline{{{{{{\rm{M}}}}}}}$ point. **b** Calculated spectral functions of single-layer ZrTe_2_ in the normal and condensed phases. The mean-field solutions of the model in the condensed phase are shown as red dashed curves.

Upon cooling to low temperatures, the topmost valence band develops a dispersionless (flat) top around the zone center in a range of ~±0.072 Å^−1^(Fig. 1f). The downward shift of the band top results in a gap of 97 meV from the Fermi level at the zone center at 10 K. This strong band renormalization is consistent with the excitonic insulator scenario that excitons consist of holes in the valence band and electrons in the conduction band condense naturally in single-layer ZrTe_2_. The flat band dispersions correspond to a zero group velocity for the electrons and holes in the condensed state. As seen in Fig. 2a, the flat valence band top at 10 K becomes much weaker in spectra taken with *p* polarized light. Another relevant feature of exciton formation is the hybridization of valence and conduction bands, which gives rise to strong folded valence bands, as observed around the $\overline{{{{{{\rm{M}}}}}}}$ point at 10 K (Fig. 2a).

### Excitonic insulator model

These observations can be understood in terms of an excitonic phase based on a BCS-like model^6^. Calculated spectral functions for the valence and conduction bands along $\overline{\Gamma {{{{{\rm{M}}}}}}}$ are shown in Fig. 2b. The results for the normal phase reproduce the essential features of the ARPES data including the spin-orbit splitting of the two valence bands. For the condensed phase, a mean-field self-consistent excitonic order parameter ∆_X_ is included in the calculation to account for, to first order, the hybridization of conduction and valence bands. The main effects are to flatten the valence band top and to open a gap of 74 meV, both in agreement with the ARPES results. The flat valence band top around the zone center gets folded to the $\overline{{{{{{\rm{M}}}}}}}$ point with considerable spectral weight transfer, which indicates a strong electronic nature of the phase transition^19^.

### Temperature dependence of the energy gap

**Fig. 3:**
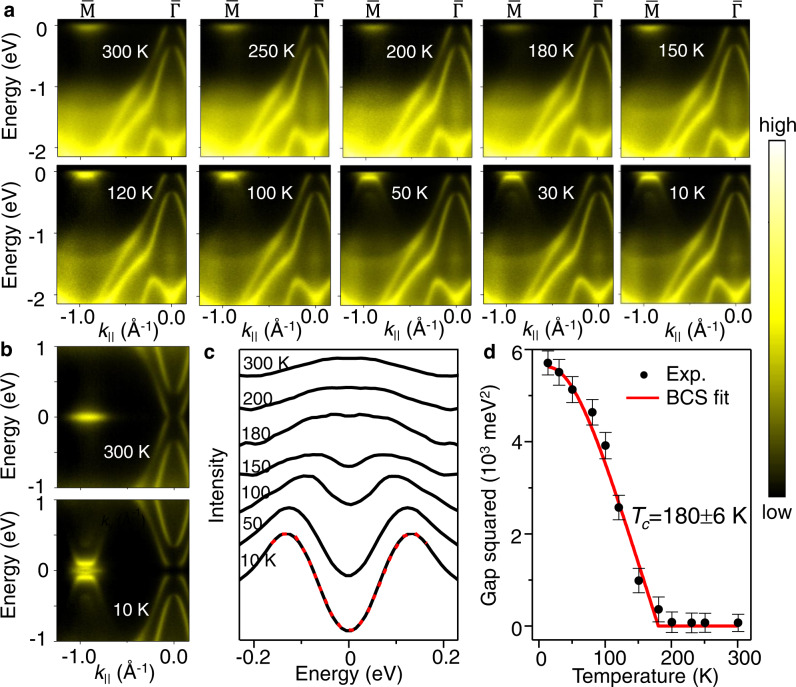
Temperature dependence of the band structure and the Bardeen–Cooper–Schrieffer (BCS)-like behavior of the energy gap. **a** ARPES spectra along $\overline{\Gamma {{{{{\rm{M}}}}}}}\,$ reveal the development of the flat valence band top and it shifts away from the Fermi level when the temperature is decreased from 300 to 10 K. The data were taken with 40 eV photons. **b** ARPES maps symmetrized in energy about the Fermi level show a gap in the condensed phase. **c** Symmetrized EDCs at the zone center at selected temperatures between 10 and 300 K. By symmetrization, the effect of Fermi–Dirac distribution at high temperatures can be canceled out. An example of the fit to a phenomenological BCS-type function^34^ is shown as a red dashed curve for the EDC obtained at 10 K. **d** The extracted temperature dependence of the square of the energy gap. The red curve is a fit using a BCS-type mean-field equation. Transition temperature *T*_C_ is labeled. The error bar is deduced from the standard deviation of the fitting.

Systematic scans of the bands along $\overline{\Gamma {{{{{\rm{M}}}}}}}$ (Fig. 3a) as a function of temperature reveal details of the development of the flat band feature, the opening of the gap, and the intensity variation of the folded bands in connection with the semimetal-insulator transition. The gap formation is illustrated by the symmetrized ARPES maps in Fig. 3b. The symmetrized energy distribution curves (EDCs) at the zone center at various temperatures are shown in Fig. 3c. At high temperatures, a single peak at the Fermi level at *E*_F_ indicates that the valence band top crosses over *E*_F_. At lower temperatures, this single peak bifurcates, indicating the opening of a gap. The energy gap, determined from the difference between the valence band top and the conduction band bottom, is 75 meV at 10 K. The square of the extracted energy gap is plotted as a function of temperature in Fig. 3d. Fitting to a BCS mean-field equation^34^ (red curve in Fig. 3d) yields a transition temperature of *T*_C_ = 180 ± 6 K.

### Charge density wave (CDW) order

The nature of the phase transition is complicated by the possibility of a Peierls-type CDW transition driven by lattice instability, as opposed to an excitonic transition driven by electron–hole interaction. We performed first-principles calculations for a ($2\times 2$) superstructure to check the effects of lattice instability (Supplementary Fig. 6). The calculated total energy per chemical unit is lower by just 0.4 meV for the ($2\times 2$) phase relative to the normal phase for single-layer ZrTe_2_. This is much smaller than the corresponding energy lowering of 5 meV for the ($2\times 2$) CDW in single-layer TiSe_2_^30^. The anharmonic well potential indicates intrinsic lattice instability in TiSe_2_, whereas the effect is not obvious in ZrTe_2_ (Supplementary Fig. 8b). Calculated atomic displacements in the CDW phase show δZr is 0.7% of the lattice constant (0.028 Å) and δTe is 0.3% of the lattice constant. As a comparison, the amplitude of δTi is 2.5% of the lattice constant (0.088 Å) in the single-layer TiSe_2_ in the CDW phase^30,35^. Although an imaginary phonon mode is found for single-layer ZrTe_2_ at the $\overline{{{{{{\rm{M}}}}}}}$ point, the PBE band structure shows no gap opening, indicating a weak lattice instability compared to the other known CDW cases (Supplementary Figs. 6 and 10).

To estimate how large the lattice distortion is necessary to open a gap of 75 meV observed by ARPES, we imposed various amounts of lattice distortion (represented by δZr) in calculations and the obtained value is ~3.15% of the lattice constant, 4.5 times of the optimized CDW structure. Hubbard U = 6 eV were further included to account for the electron localization and the gap remains closed in the CDW phase where δZr = 0.7% of the lattice constant. (Supplementary Fig. 8c, d). These findings indicate the insufficient ability of the lattice distortion to open a significant gap in this system, although it may contribute to the formation of the backfolded bands at the $\overline{{{{{{\rm{M}}}}}}}$ points as in the case of single-layer TiTe_2_ (δTi = 1.0% of the lattice constant)^36^. Effects of electron–hole hybridization can be better described using the HSE functional instead of the PBE in the calculation. Similar to the mean-field self-consistent method mentioned above, HSE calculations yield a gap of ~10 meV near the $\overline{\Gamma }$ point, indicating the necessity of the electron–hole interaction in this system. Of note is that DFT results predict a much larger overlap between the valence band top and the conduction band bottom than the experiment in the normal phase. The absolute value of the quantity predicted by the calculations may not be accurate due to the inherent limitations of the DFT method. However, the relative comparison between TiSe_2_ and ZrTe_2_ should remain valid.

The structural distortion in a single-layer film can be revealed by low-energy electron diffraction (LEED). The elastic scattering of the incident electrons with the crystal potential mostly comes from the core electrons and nuclei of crystals^37^. LEED patterns of single-layer TiSe_2_ show the 1/2 order diffraction spots corresponding to the ($2\times 2$) CDW phase at low temperatures (Supplementary Fig. 9a, b). However, no CDW diffraction spots were observed for single-layer ZrTe_2_ in the probed beam energy range (20–200 eV), indicating the small lattice distortion in the CDW phase. Consistent results were obtained for different batches of samples. As a first-order approximation, the proportion of CDW spot intensity (I_CDW_) to the corresponding Bragg spot intensity (I_Bragg_) is proportional to the square of the atomic displacements (μ) in the CDW phase^38^. With background intensity subtracted, I_CDW_/I_Bragg_ for single-layer TiSe_2_ is ~0.3. The noise level divided by the saturated intensity is used to establish the detection limit and the value is ~0.05, indicating μ_ZrTe2_ should be less than 2/5 of μ_TiSe2_ on average (Supplementary Fig. 9c–e), in consistent with the above prediction that μ_ZrTe2_ ~ μ_TiSe2_/3. Evidently, our LEED results set the upper bound of the atomic displacement of single-layer ZrTe_2_ in the CDW phase and the phase transition in this material cannot be described in terms of the usual Peierls-type CDW.

### Dimensionality and carrier screening effect

**Fig. 4:**
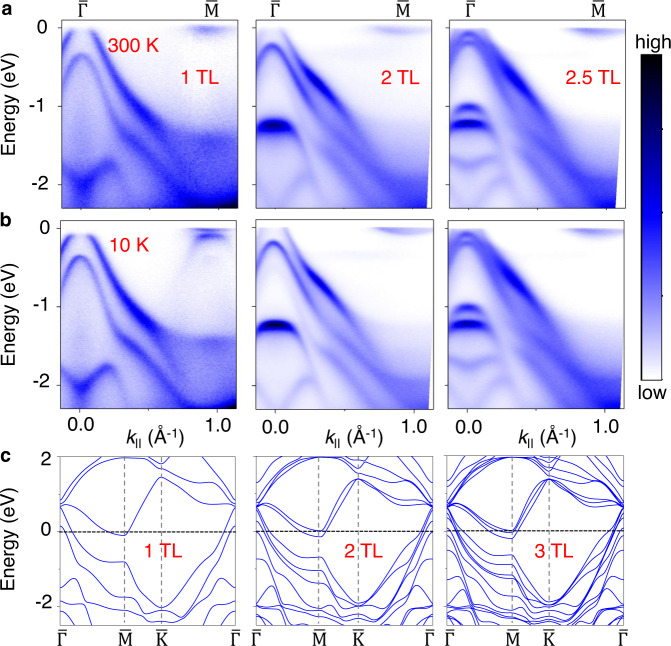
Thickness dependence of the band structure. ARPES maps along $\overline{\Gamma {{{{{\rm{M}}}}}}}$ for *N*-layer ZrTe_2_ (*N* = 1, 2, 2.5) taken with unpolarized light (21.2 eV) at **a** 300 K and **b** 10 K. **c** Calculated band dispersions (*N* = 1, 2, and 3) for the normal phase.

Since no exciton condensation occurs in bulk ZrTe_2_, an interesting question is how such a transition evolves with the sample thickness from the 2D limit of a single layer to the bulk limit. ARPES spectra for *N* = 1, 2, 2.5 trilayer (TL) ZrTe_2_ taken with the unpolarized light from a He-discharge lamp along the $\overline{\Gamma {{{{{\rm{M}}}}}}}$ direction are presented in Fig. 4 (the *N* = 2.5 case represents a mixture of *N* = 2 and 3). The measured dispersion relations at 300 K and the multiplication of bands as *N* increases are in excellent agreement with theoretical results for the normal phase. All cases are metallic in the normal phase, with bands crossing the Fermi level. However, only the 1 TL sample shows gap opening and valence band folding at 10 K. Thus, the excitonic phase in the single layer is suppressed by adding just one TL. This suppression can be attributed to changes in the carrier density, which can be extracted from the Luttinger area of the Fermi surface^39^. The carrier density around the $\overline{{{{{{\rm{M}}}}}}}$ point for a 2 TL film is ~4.8 × 10^13^/cm^2^, which is 136% larger than the 1 TL case. The extra carrier density leads to a stronger screening between the electrons and holes, thus hampering the formation of the excitons. A very weak CDW order may still exist in multilayer films^40^, but it does not lead to an excitonic phase.

To further explore the effects of carrier screening, we have employed in situ surface doping of K on the 1 TL sample (Supplementary Fig. 1a). With the sample at 10 K, increasing amounts of K doping cause the valence band top to move upward and poke through *E*_F_ at a critical carrier density of 3.8 × 10^13^/cm^2^. The folded valence bands also disappear at this critical doping level. The lower valence band, separated by spin-orbit splitting from the top valence band, also shifts up slightly upon initial K doping. However, further increasing the doping level beyond the critical value causes it to shift downward together with the conduction band. Thus, the overall movements of the bands cannot be described by a simple rigid shift of all bands, other effects like atom intercalation can renormalize the band structure^41^. An implication is that electron doping leads to the suppression of the bound electron–hole pairs; beyond the critical doping level, the gapped ground state is destroyed, and the system returns to a normal semimetallic state.

The total evidence based on both experiment and theory suggests that single-layer ZrTe_2_ is a prototypical 2D excitonic matter. A strong interband electron–hole coherent coupling in 2D and poor electronic screening by a low carrier density around the Fermi level conspire to make this system a unique case to display the requisite flat bands for excitonic condensation. The results are relevant to the development of a comprehensive understanding of the physics of exciton condensation in solids. The high transition temperature in this case suggests a versatile platform for experimentation in quantum effects and phenomena based on exciton coherence.

## Methods

### ARPES measurements

ZrTe_2_ thin films were grown in situ in the integrated MBE/ARPES systems at the laboratory at Shanghai Jiao Tong University, beamlines 10.0.1 at Advanced Light Sources in Lawrence Berkeley National Laboratory, and beamline 13U at National Synchrotron Radiation Lab in Hefei. Samples were transferred via a vacuum suitcase to beamline 03U at Shanghai Synchrotron Radiation Facility^42^. Substrates of 4H-SiC were flash-annealed for multiple cycles to form a well-ordered bilayer graphene on the surface. Single-layer and multilayer ZrTe_2_ were grown on top of the substrate by co-evaporating high-purity Zr and Te from an electron-beam evaporator and a Knudsen effusion cell, respectively, while the substrate was maintained at 600 °C. The growth process and thickness of the films were monitored by RHEED, and the growth rate was set to 60 min per layer of ZrTe_2_. The formation of the multilayer of ZrTe_2_ is also evidenced by the evolution of the band structure measured by ARPES. ARPES measurements were performed at a base pressure of ~5 × 10^−^^11^ mbar with an in-laboratory He-discharge lamp (He-I 21.2 eV) and 16–100 eV photons at synchrotron using Scienta DA30 analyzers. The energy resolution is better than 15 meV and the angular resolution is around 0.2°. Each sample’s crystallographic orientation was precisely determined from the symmetry of constant-energy-contour ARPES maps.

### Computational details of density functional theory from first principles

Calculations were performed using the Vienna ab initio package (VASP)^43,44^ with the projector augmented wave method^45,46^. A plane-wave energy cutoff of 520 eV and a $24\times 24\times 1$ *k*-mesh were employed. The generalized gradient approximation (GGA) with the Perdew–Burke–Ernzerhof (PBE) functional^47^ was used for structure optimization of the single-layer ZrTe_2_. Freestanding films were modeled with a 16-Å vacuum gap between adjacent layers in the supercell. The fully optimized in-plane lattice constant for a single-layer 1*T*-ZrTe_2_ is *a* = 3.974 Å. Since PBE functional is known to underestimate band gap, HSE functional^48^ was also employed to better capture electron–hole hybridizations. For the 2 TL and 3 TL, the PBEsol functional^49^ was used to relax interlayer distance; the in-plane lattice constant was taken from the monolayer results. Spin-orbit couplings are included in the calculations. Phonon calculations were carried out using the supercell method as implemented in the Phonopy package^50^. The band unfolding was performed using the BandUP code^51,52^.

### Self-consistent mean-field theory

We study the excitonic insulator phase based on the mean-field calculation of an 11-band model. The noninteracting part of the model Hamiltonian was constructed from wannierized orbitals^53^ of first-principle HSE calculations. ${{{{{{\rm{H}}}}}}}^{0}=\sum {{{{{{\rm{\epsilon }}}}}}}_{{{{{{\rm{nk}}}}}}}{c}_{{nk}}^{{{\dagger}} }\,{c}_{{nk}}$. We consider interactions between two topmost valence bands and two lowest conduction bands. The interaction part of the Hamiltonian reads

1 $${{{{{{\rm{H}}}}}}}^{{{{{{\rm{int}}}}}}}=\frac{1}{N}\mathop{\sum }\limits_{{{{{{\rm{k}}}}}},{{{{{{\rm{k}}}}}}}^{{\prime} },{{{{{\rm{q}}}}}}}{{{{{\rm{V}}}}}}\left({{{{{\rm{q}}}}}}\right){c}_{c{{{{{\boldsymbol{k}}}}}}}^{{{\dagger}} }\,{c}_{c{{{{{\boldsymbol{k}}}}}}+{{{{{\boldsymbol{q}}}}}}}{c}_{v{{{{{{\boldsymbol{k}}}}}}}^{{\prime} }}^{{{\dagger}} }\,{c}_{v{{{{{{\boldsymbol{k}}}}}}}^{{\prime} }-{q}^{{\prime} }}$$

where $N$ is the number of *k*-points in the Brillouin zone (BZ). We assume that the interaction does not depend on band indices and crystal momentum $k$ and ${k}^{{\prime}}$ and takes the form of screened Coulomb interaction ${{{{{\rm{V}}}}}}\left({{{{{\rm{q}}}}}}\right)=\frac{{{{{{\rm{U}}}}}}{{\tanh }}\frac{\xi q}{2}}{{{{{{\rm{\xi }}}}}}{{{{{\rm{q}}}}}}/2},$^24^ where ${{{{{\rm{\xi }}}}}}$ is set to 25 nm and U is a tuning parameter. To solve the excitonic insulator state, we consider a set of order parameters of electron–hole coherence ${\Delta }_{X}({{{{{\bf{Q}}}}}})=\frac{1}{N}{\sum }_{{{{{{\rm{k}}}}}}}\langle {c}_{{ck}}^{{{\dagger}} }{c}_{{vk}+Q}\rangle$ with wavevectors ${{{{{\bf{Q}}}}}}={{{{{{\bf{b}}}}}}}_{1}/2$, $-{{{{{{\bf{b}}}}}}}_{2}/2$, and $({{{{{{\bf{b}}}}}}}_{2}-{{{{{{\bf{b}}}}}}}_{1})/2$, where ${{{{{{\bf{b}}}}}}}_{1}$ and ${{{{{{\bf{b}}}}}}}_{2}$ are two in-plane reciprocal lattice vectors. These wavevectors correspond to the triple-q CDW states observed in the experiment^40^.

We numerically solved the Hamiltonian in a folded BZ with a uniform sampling of 48 × 48 *k*-grid in the BZ. A *k*-point in the full BZ is labeled by a corresponding *k*-point in the folded BZ and a wave vector connecting them. We found that with a parameter ${{{{{\rm{U}}}}}}=65$, the result agrees well with the experiment. The self-consistent calculation is initialized with a nonzero order parameter then the mean-field Hamiltonian is diagonalized. From the eigenvectors, we can compute the new order parameters and repeat the procedure until convergence. The spectral function is computed following ref. ^54^. We have

2 $${{{{{\rm{A}}}}}}\left({{{{{\rm{k}}}}}},{{{{{\rm{\omega }}}}}}\right)=\mathop{\sum }\limits_{{{{{{\rm{n}}}}}}}\mathop{\sum }\limits_{{{{{{\rm{j}}}}}}}\left|{{{{{{\rm{\varphi }}}}}}_{{{{{{\rm{nk}}}}}}}^{{{{{{\rm{j}}}}}},{{{{{{\rm{q}}}}}}}_{0}}\left({{{{{\rm{k}}}}}}\right)}\right|^{2}{{{{{\rm{\delta }}}}}}\left({{{{{\rm{\omega }}}}}}-{{{{{{\rm{E}}}}}}}_{{{{{{\rm{nk}}}}}}}\right),$$

where ${{{{{{\rm{E}}}}}}}_{{{{{{\rm{nk}}}}}}}$ and ${{{{{{\rm{\varphi }}}}}}}_{{{{{{\rm{nk}}}}}}}^{{{{{{\rm{j}}}}}},{{{{{{\rm{q}}}}}}}_{0}}$ are the n-th eigenenergy and eigenfunctions of the mean-field Hamiltonian with momentum k, respectively, j is the index of the bare band and ${{{{{{\rm{q}}}}}}}_{0}=0$ labels components from the folded BZ centered at $\overline{\Gamma }$ since the momentum k in the spectral functions is defined in the unfolded zone.

## Supplementary information

Supplementary Information

## Data Availability

General methods, experimental procedures, and characterizations are available within the article and the Supplementary Information. Other relevant data are available from the corresponding author upon reasonable request.
