# Supplementary material for: Evidence of high-temperature exciton condensation in a two-dimensional semimetal
Source: Nat Commun. 2023 Feb 22;14:994. doi: 10.1038/s41467-023-36667-x (PMC9946959; doi:10.1038/s41467-023-36667-x)
Supplement: Supplementary file 1 — Supplementary Information [file 41467_2023_36667_MOESM1_ESM.pdf]

Evidence of high-temperature exciton condensation in a two-dimensional semimetal

Supplementary Note 1. Valence band dispersions and fits for the single-layer $ZrTe_2$

Supplementary Figs. 2a and 2b show ARPES spectra taken with *s* polarized photons at 300 K and 10 K along the $\overline{\Gamma M}$ direction, respectively, overlaid with the band dispersion relations extracted from data fitting. Since the outer valence band crosses over the Fermi level and ARPES measurements do not reveal the states above the Fermi level, we use a quadratic function to fit the dispersion and obtain the band top assuming noninteracting states.

$$E_v(\boldsymbol{k}) = E_{v0} + \frac{\hbar^2}{2m_v^*}k^2 \qquad (3)$$

where E_{v0} is the energy of valence band top and m_v^* is the effective mass. The fitting results and dispersion relations are plotted together in Supplementary Fig. 2c. The valence band maximum in the normal phase is 37 meV above the Fermi level, which agrees well with the value from the tight-binding model. The renormalization of valence band is evident in the condensed phase. With excitonic coupling, the valence bands shift to the deeper binding energies by 26 meV and the band top flattens.

Supplementary Note 2. Determination of conduction band peak positions

The ARPES spectra taken with *p* polarized photons at 10 K along the $\overline{\Gamma M}$ direction have been shown in Supplementary Fig. 4a. It is difficult to determine the conduction band position accurately since only the band edge is shown around the Fermi level. We extract the band position by fitting to the EDCs at $\overline{M}$ point with Lorentzian peaks multiplied by the Fermi-Dirac function and convoluted with a Gaussian corresponding to the experimental energy resolution (15 meV), as shown in Supplementary Fig. 4b. A Shirley background is subtracted to remove the effect of

secondary electrons. The full width at half maximum (FWHM) of the conduction band peak is 46 meV at 10 K, only half of the value of the valence band peak (103 meV). This result suggests that only part of the conduction band crosses over the Fermi level and the band gap determined in the main text is underestimated.

Supplementary Note 3. Calculated band structure and CDW instability for single-layer $ZrTe_2$

Transition metal dichalcogenides with indirect gap tends to induce the formation of CDW state with a spanning vector that connects the valence band maximum to the conduction band minimum. A further test for CDW effect in single-layer $ZrTe_2$ is provided by calculating the bands in (2×2) superstructure. The computed band structures of (1×1) lattice in the normal phase and (2×2) in the CDW phases are shown in Supplementary Fig. 6. The results with PBE functional agree well with the ARPES in the normal phase, although the overlap between valence and conduction bands is overestimated in the calculations (0.7 eV). We note that there is a band inversion between Zr *d* and Te *p* states at the $\bar{\Gamma}$ point around 0.6 eV above the Fermi level in the normal phase, similar to the previous report and the bulk case, which suggests a topological nontrivial character[1,2]. However, the valence band top separates from the conduction band and no band inversion were observed with HSE functional, in consistent with the MBJ calculations[3].

Supplementary Note 4. Spectral weight projection

Supplementary Fig. 11a shows the spectral functions of single-layer $ZrTe_2$ in the condensed phase. In order to understand how the bands evolve from the normal phase to the condensed phase, we project the spectral weight of the bands in the condensed phase onto the top valence band and

bottom conduction band in the normal phase, as shown in Supplementary Fig. 11b and 11c, respectively. The results indicate the spectral weight of the valence band top around $\bar{\Gamma}$ has a different origin from the backfolded band top around $\bar{M}$. The former is primarily derived from Te *p* states and the latter is primarily derived from Zr *d* states, which explains the spectral weight difference in these two bands in different polarization measurements.

Supplementary Note 5. Matrix element effect

We did symmetry analysis on the transition matrix element. The interaction Hamiltonian can be simplified as $\frac{e}{m_e c}$A·P within the dipole approximation, where A is the vector potential of an electromagnetic radiation and P is the electron momentum operator. The photoemission intensity is proportional to the dipole transition matrix element $< \psi_f|\mathrm{A}\cdot\mathrm{P}|\psi_i >$, where $|\psi_i >$ and $|\psi_f >$ are the initial and final states, respectively. First-principles calculations yield the initial state and the final state of photoemission is approximated as a plane wave. The experimental setup is shown in the Supplementary Fig. 12a. Vector potential of photons are $A_p = (A_x, 0, A_z)$ and $A_s = (0, A_y, 0)$ for *p* and *s* polarizations, respectively. The selection rule for *p* and *d* orbitals are summarized in the Table (Supplementary Fig. 12d)[4].

The projected orbitals from the DFT calculations show the p_x and p_y dominate the valence band top around the $\bar{\Gamma}$ point. For *p* polarization spectra, only p_x orbital is allowed and its contribution to the intensity is small. It is only 25% considering a 45° angle between A_p and the A_x axis, which explains the vanishing intensity of the valence band top, as shown in the Supplementary Fig. 12c. The conduction and folded bands around the $\bar{M}$ point are dominated by d_{xz}, d_{yz}, and d_{z^2} orbitals and they can only be revealed by the *p* polarized light. It should be noted that this analysis only

qualitatively reveals the matrix element effect since the intensity of an orbital can also be affected by the cross section of the photoelectron.

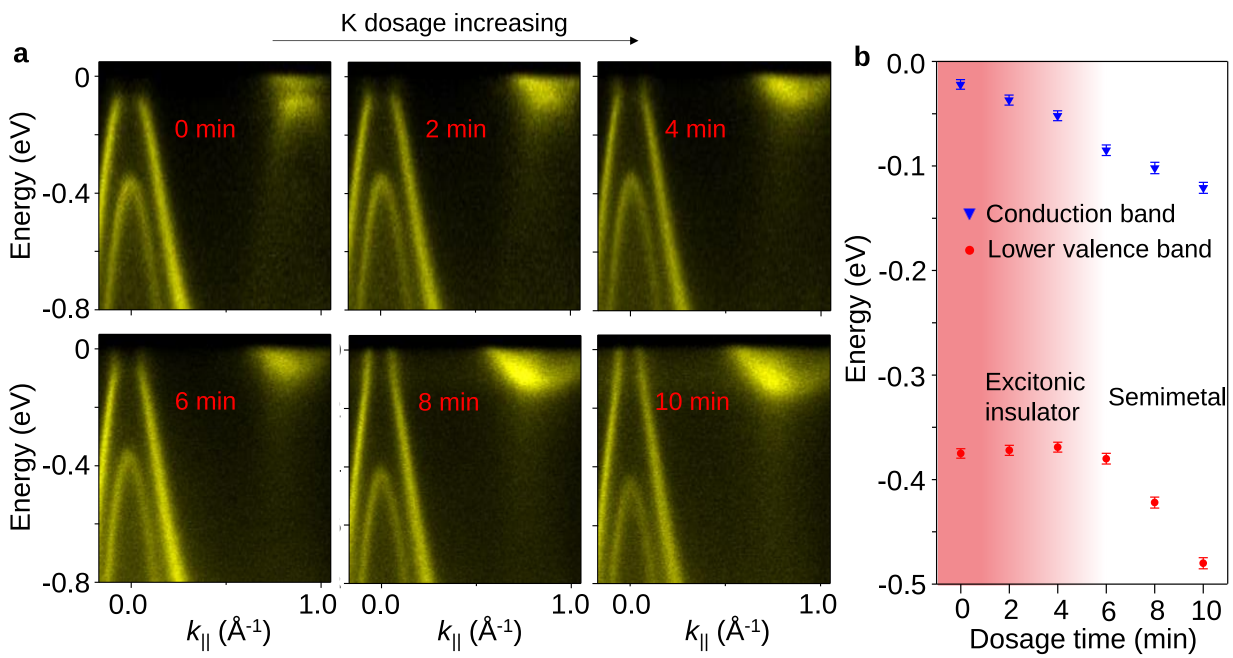

Supplementary Fig. 1 | K surface doping on the single-layer $ZrTe_2$. (**a**) ARPES maps taken along $\overline{\Gamma M}$ direction at 10 K. The total time of the dosage is labeled. (**b**) The extracted lower valence band and conduction band energy as a function of the dosage time. The error bar is deduced from the standard deviation of the fitting.

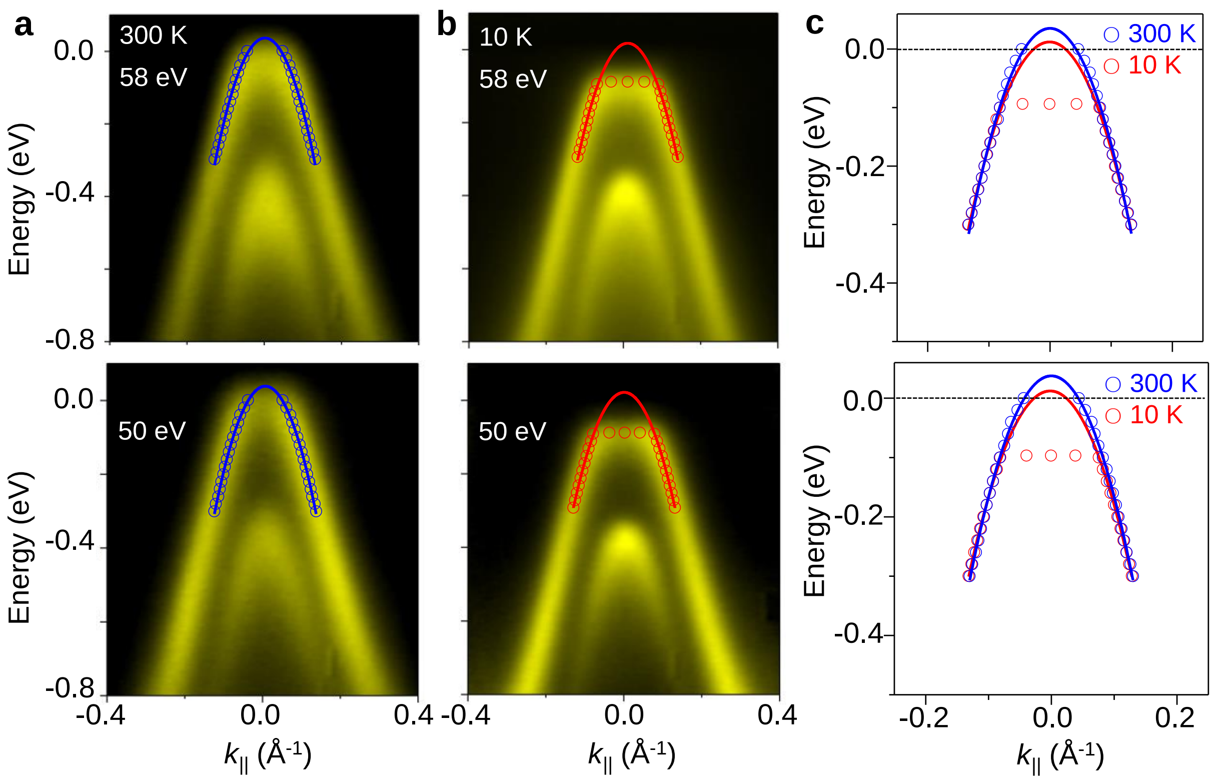

Supplementary Fig. 2 | Valence band dispersions for the single-layer $ZrTe_2$. ARPES maps taken with *s* polarized photons (50 and 58 eV) along $\overline{\Gamma M}$ direction at (**a**) 300 K in the normal phase and (**b**) 10 K in the condensed phase. Blue and red circles indicate the dispersions extracted from fitting to the EDCs/MDCs of the ARPES data at 300 K and 10 K, respectively. The blue/red curves are quadratic fits to the peak positions of the bands, indicating the dispersions without the excitonic coupling. (**c**) The extracted ARPES dispersions and fitting results plotted together for a comparison.

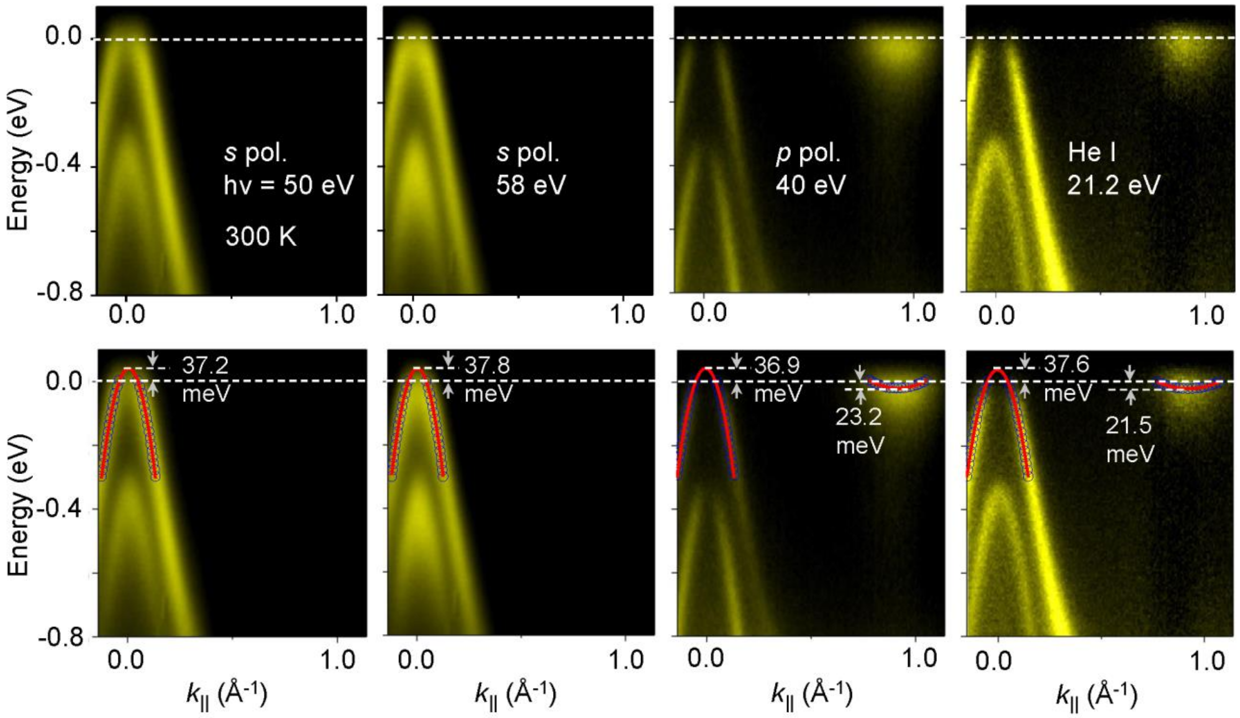

Supplementary Fig. 3 | ARPES spectra with varied photon energies and polarizations. ARPES maps taken with *s*, *p*, and unpolarized photons along $\overline{\Gamma M}$ direction at 300 K in the normal phase. The spectra in the lower row were overlaid with band dispersions extracted from data fitting. A quadratic function was used to fit the valence/conduction band dispersions and similar band top/bottom positions were obtained.

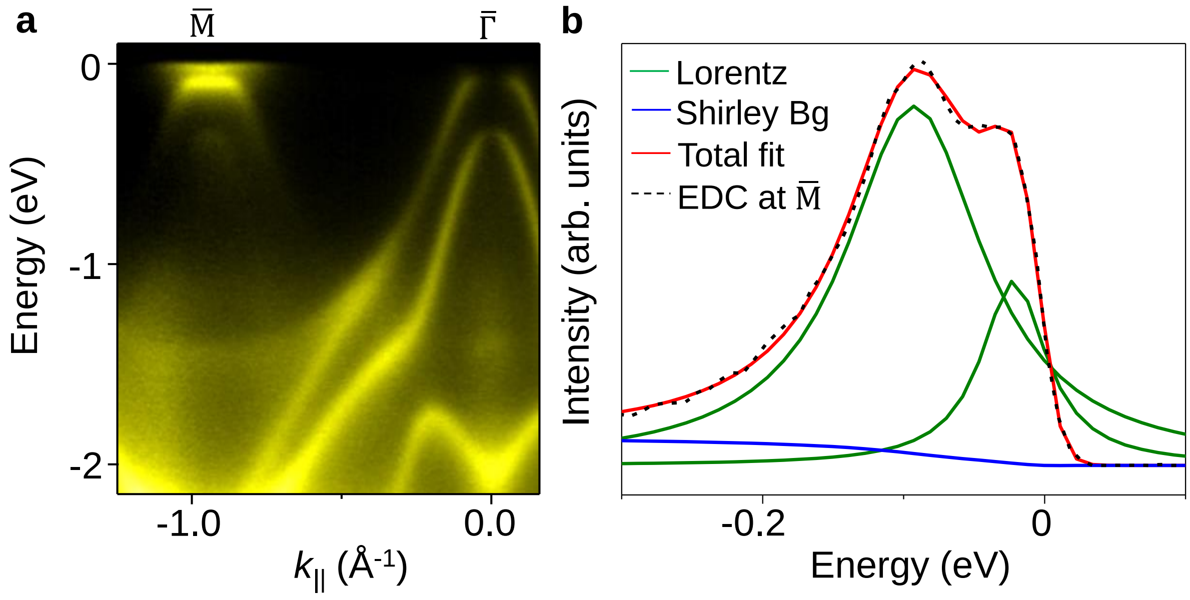

Supplementary Fig. 4 | Extraction of conduction band peak positions. (**a**), ARPES maps taken with *p* polarized photons along $\overline{\Gamma M}$ direction at 10 K in the condensed phase. Examples of fit to the EDCs at the $\overline{M}$ point in these phases are shown in panels (**b**). In the condensed phase, two Lorentzian peaks are used to represent the conduction and backfolded valence band peaks. The red curves are the fitting results which are a combination of the Shirley background, the Lorentzian peaks multiplied by the Fermi-Dirac function and convoluted with a Gaussian corresponding to the experimental energy resolution.

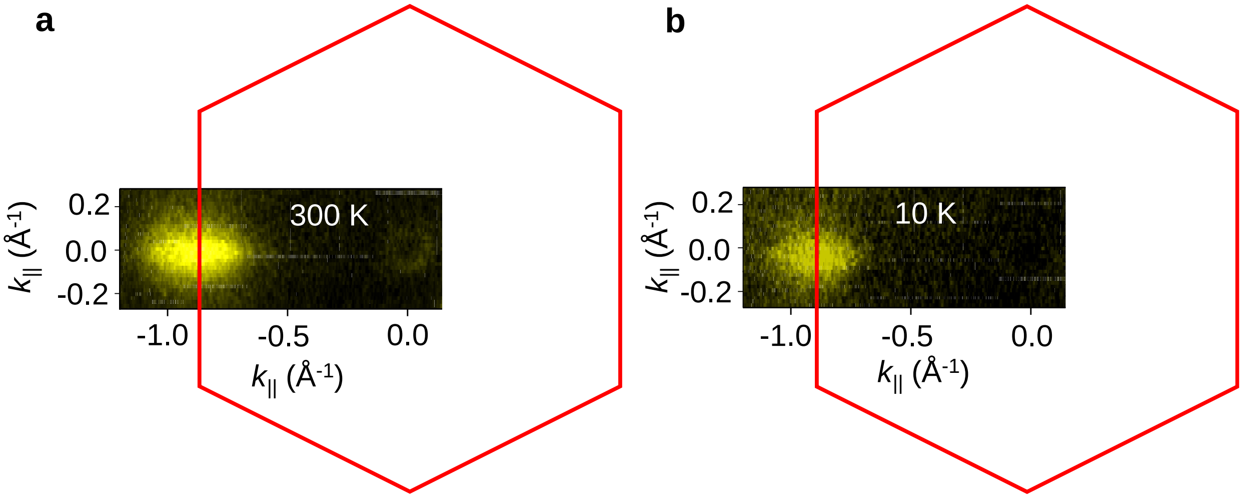

Supplementary Fig. 5 | Fermi surface of single-layer $ZrTe_2$ in the normal and condensed phases. Measured Fermi surface maps in (**a**) the normal phase at 300 K and (**b**) the condensed phase at 10 K obtained by integrating the ARPES intensity over ±10 meV about the Fermi level. The red hexagons indicate the first Brillouin zone. The experiment contour in the normal phase consists of electron pockets of eclipse shape centered around the $\bar{M}$ points and a hole pocket of circle shape around the zone center. In the condensed phase, the hole pocket segment disappears due to the gap opening around the zone center.

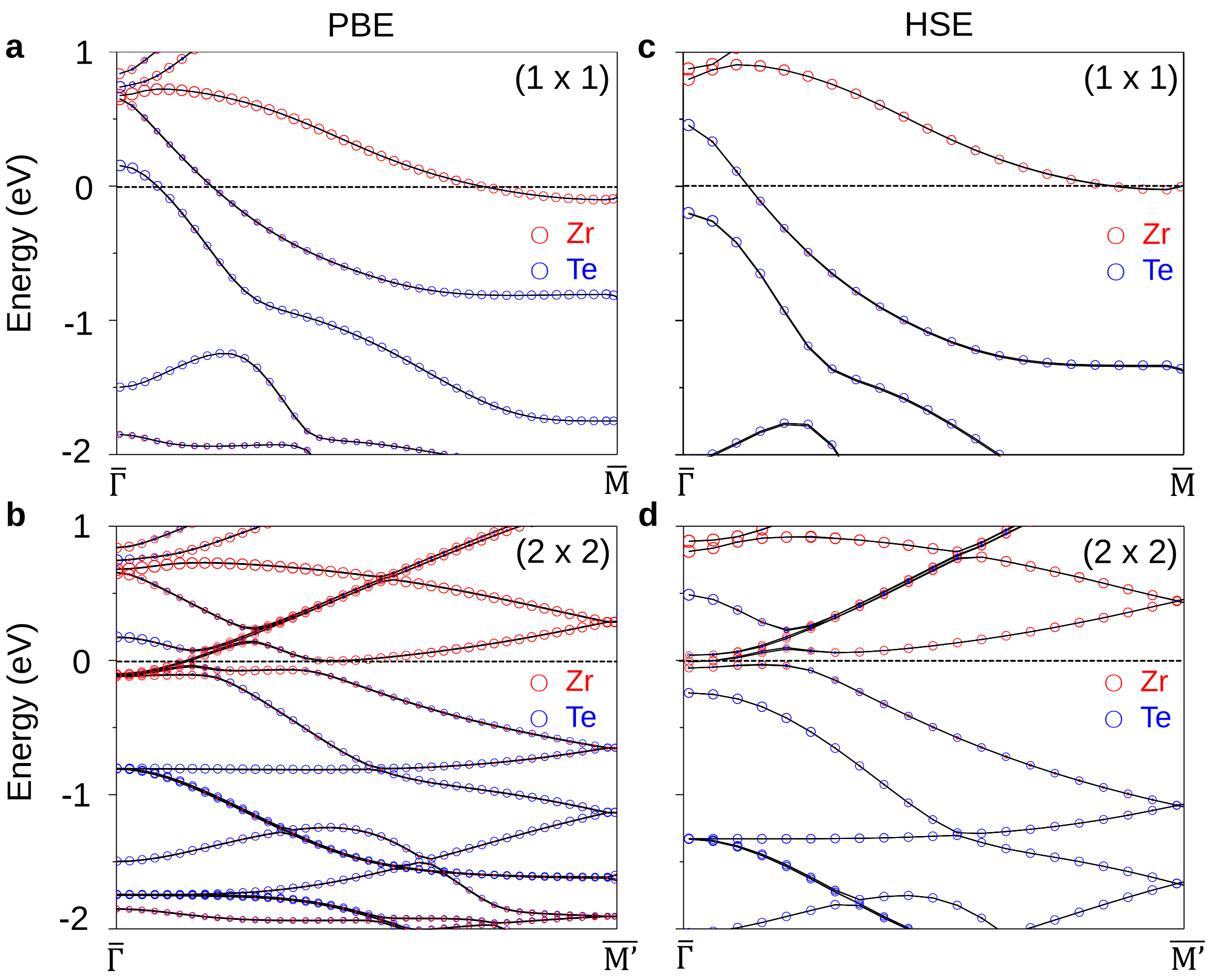

Supplementary Fig. 6 | DFT band dispersions for the (1 × 1) and (2 × 2) structures of single layer $ZrTe_2$. Calculated band structures for (**a**) (1 × 1) in the normal phase and (**b**) (2 × 2) superstructure using the PBE method. The system remains a semimetal in the (2 × 2) superstructure as the bands cross over the Fermi level. (**c**) and (**d**), band dispersions for the same structure using the HSE functional. A small gap opens around the $\bar{\Gamma}$ point for the (2 × 2) structure. The band characters, Zr 4*d* or Te 5*p*, are color coded. $\overline{M'}$ is in the middle of $\overline{\Gamma M}$ in the (1 x 1) structure.

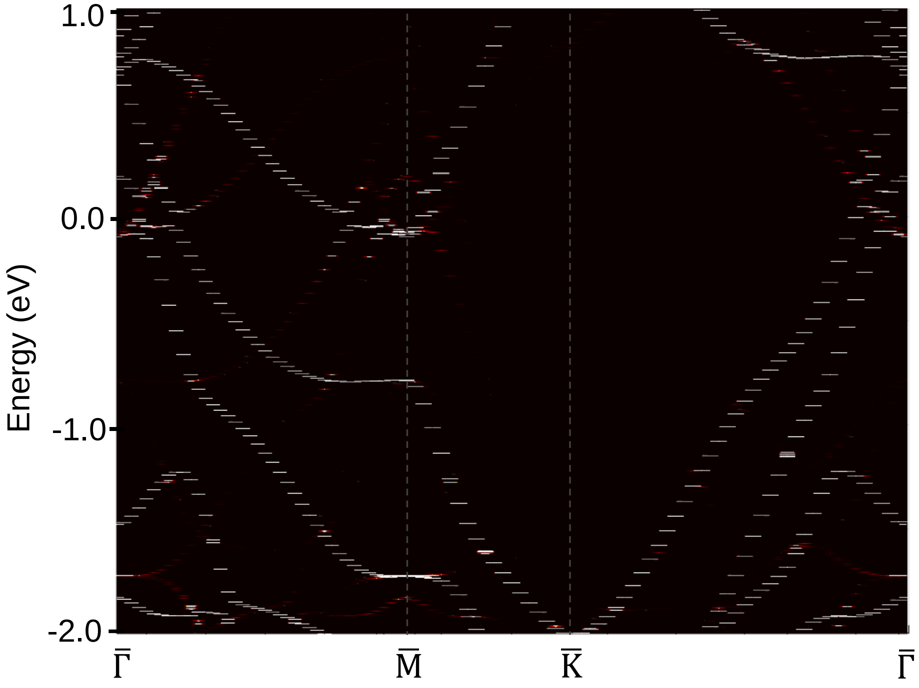

Supplementary Fig. 7 | Unfolded band dispersions for the (2 × 2) structure of single layer $ZrTe_2$. Calculated unfolded band structure for (2 × 2) superstructure using the PBE method. Weak folded bands appear around the $\bar{M}$ point and partial gaps around the Fermi level are observed.

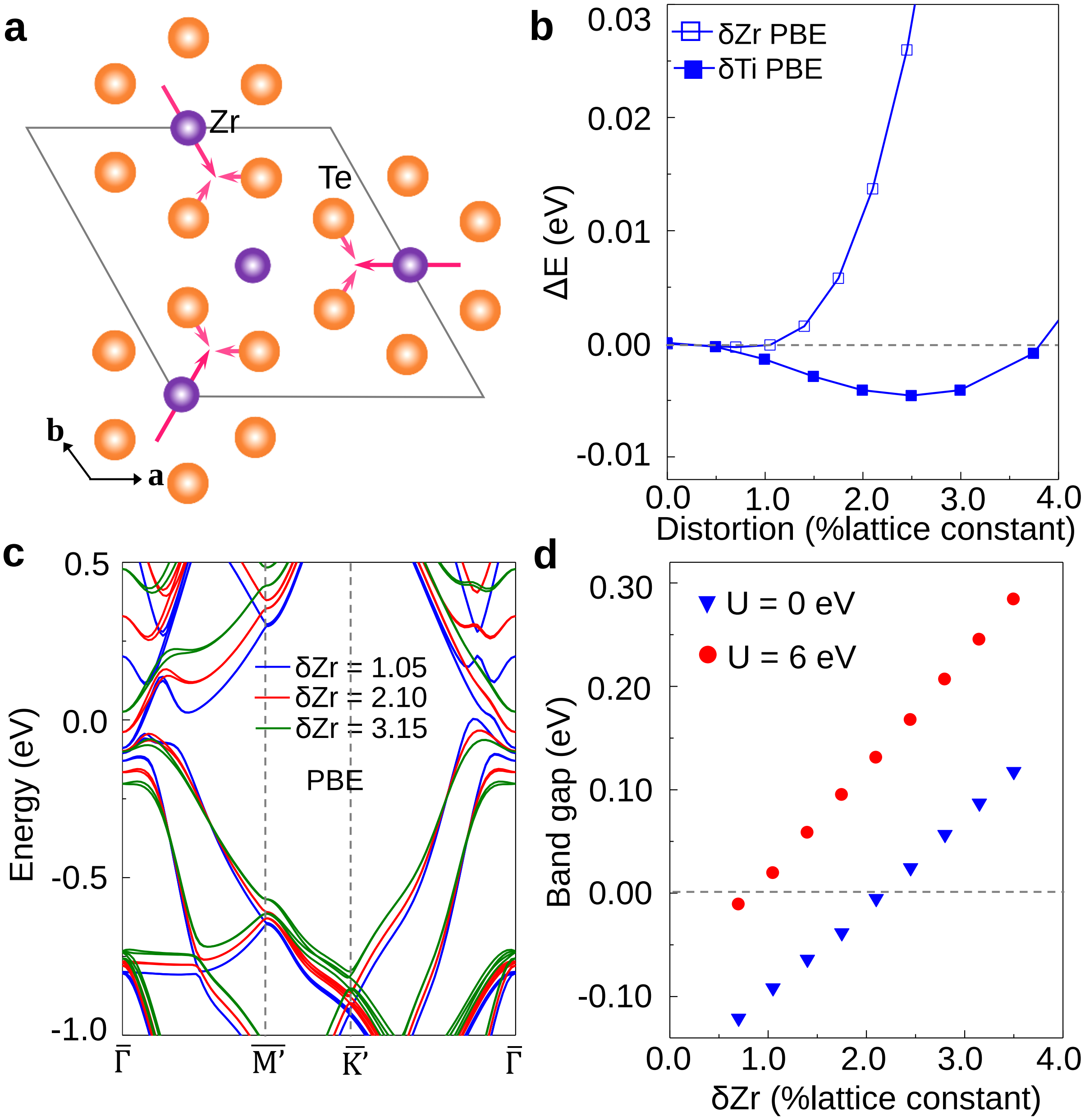

Supplementary Fig. 8 | Calculated atomic displacement, lattice distortion and corresponding band gap. (**a**), Atomic displacement pattern in a (2 × 2) unit cell of single layer $ZrTe_2$. The red/pink arrows indicate computed Zr/Te atomic displacements amplified by a factor of 100. (**b**), Energy gain with respect to the normal phase as a function of δZr (open squares). The results of δTi in single layer $TiSe_2$ are also shown (solid squares) as a comparison. (**c**), Calculated band

structures for a (2 $\times$ 2) superstructure using the PBE functional with imposed different lattice distortions. The Zr displacements are labelled. (**d**), Extracted band gap for U = 0 and 6 eV as a function of the lattice distortion. Hubbard U = 6 eV is chosen by fitting the band results with the ARPES spectra in the normal phase and it is also close to the value reported for the bulk $ZrTe_2$[5]. The value of negative gap is defined as overlap between conduction band minimum and valence band maximum. The gap remains closed for both U = 0 and 6 eV with a Zr displacement of 0.7% of the lattice constant.

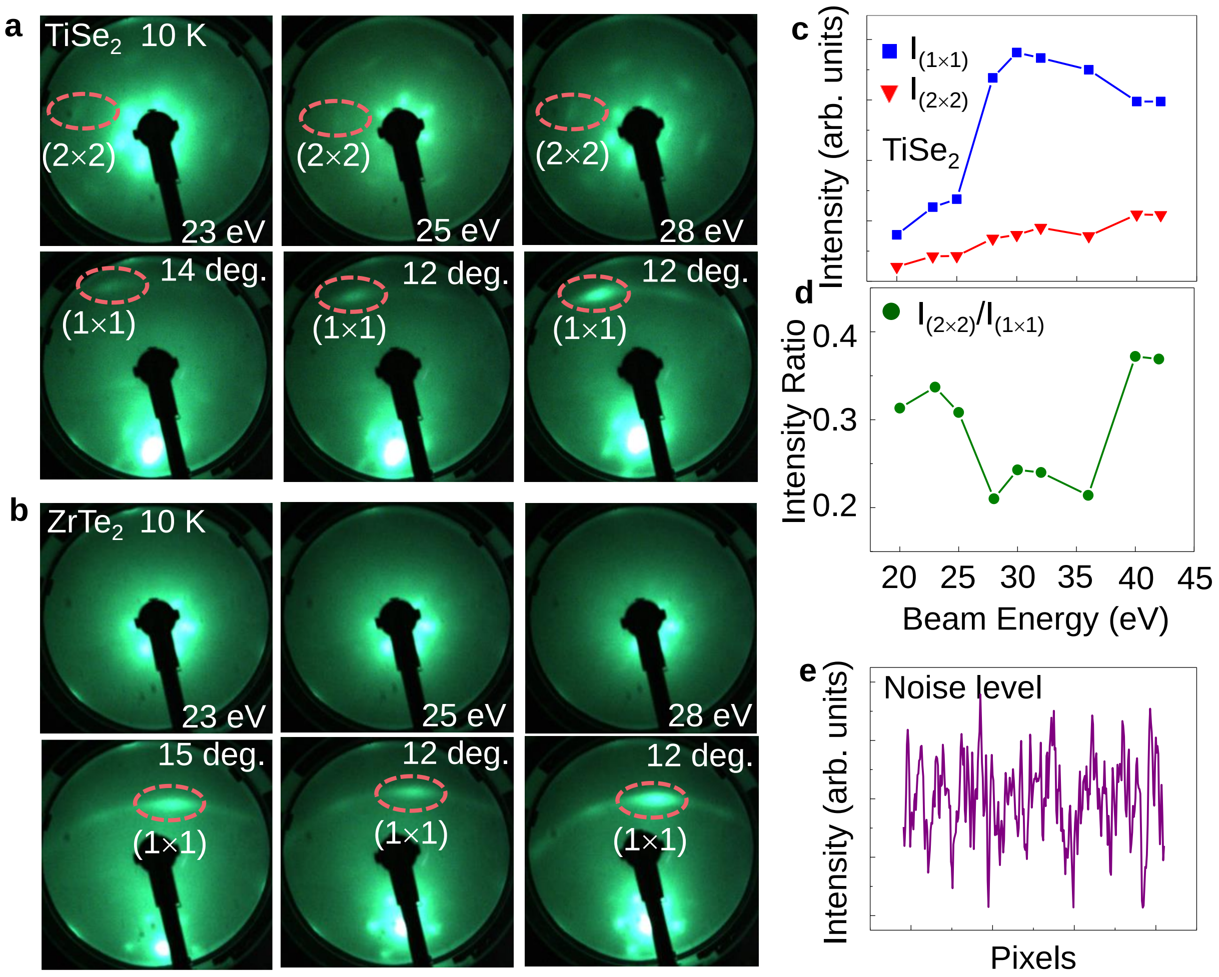

Supplementary Fig. 9 | LEED results for single layer $TiSe_2$ and $ZrTe_2$. LEED patterns of (**a**) single layer $TiSe_2$ and (**b**) $ZrTe_2$ at 10 K with selected beam energies. (**c**) Intensity of the (1×1) Bragg spots and the (2×2) CDW spots with background intensity subtracted as a function of beam energy for single layer $TiSe_2$. (**d**) Intensity ratio of the CDW spots to the Bragg spots. (**e**) An example of the noise level in the LEED measurements determined from the variation of background intensity.

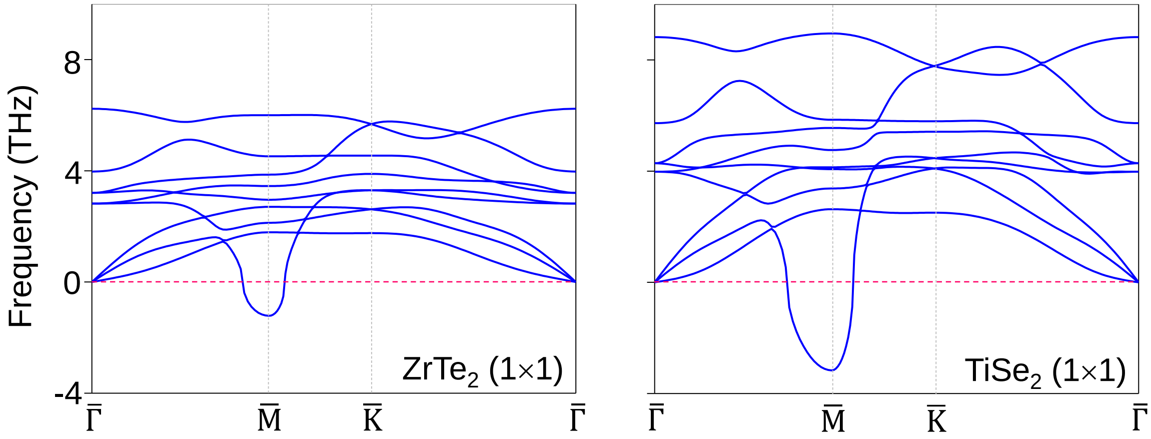

Supplementary Fig. 10 | Phonon dispersions. Calculated phonon dispersions for single layer $ZrTe_2$ and $TiSe_2$ in the normal phase. More pronounced imaginary mode at the $\bar{M}$ point indicates stronger lattice instability in single layer $TiSe_2$.

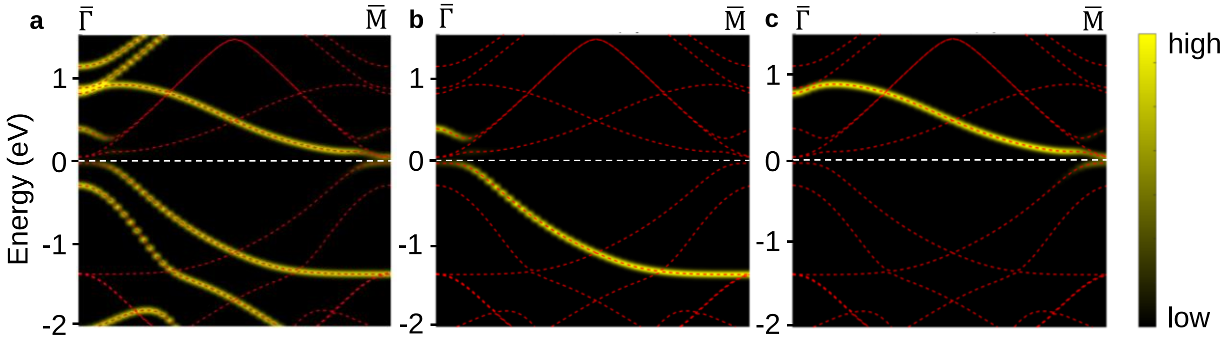

Supplementary Fig. 11 | Projected spectral weight. (**a**), Spectral functions of single-layer $ZrTe_2$ in the condensed phase. Spectral weight projection on the (**b**) top valence band and (**c**) bottom conduction band in the normal phase. The results indicate the spectral weight of valence band top is primarily from Te *p* states and backfolded band top is primarily derived from Zr *d* states. The mean field solutions of the model are shown as red dashed curves.

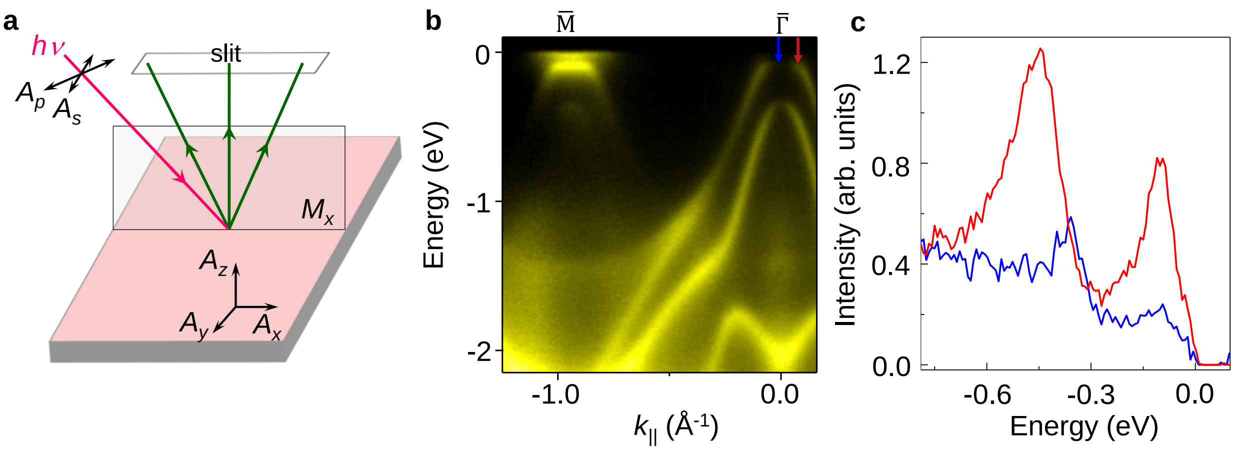

d

Polarization	Orbitals contribute to the intensity	
A_x	p_x	d_{z^2}, $d_{x^2-y^2}$
A_y	p_y	d_{xy}
A_z	p_z	d_{xz}

Supplementary Fig. 12 | Orbital character of the bands and the matrix element effect. (**a**), Experimental geometry for the ARPES measurements. M_x is the incident plane and the slit is along in the A_x direction. (**b**) The spectrum along $\overline{\Gamma M}$ direction taken at 10 K with *p* polarized light for single layer $ZrTe_2$. (**c**) The EDCs taken at the positions indicated by the arrows in (**b**). The spectral weight at the $\bar{\Gamma}$ point is suppressed because of the matrix element effect. (**d**) Table lists the orbitals contributions to the spectrum intensity for different light polarizations.

Supplementary References

1. Kar, I. et al. Metal-chalcogen bond-length induced electronic phase transition from semiconductor to topological semimetal in ZrX_2 (X = Se and Te). *Phys. Rev. B* **101**, 165122 (2020).

2. Tsipas, P. et al. Massless Dirac fermions in $ZrTe_2$ semimetal grown on InAs(111) by van der Waals epitaxy. *ACS Nano* **12**, 1696-1703 (2018).

3. Tang, F., Po, H. C., Vishwanath, A. & Wan, X. Comprehensive search for topological materials using symmetry indicators. *Nature* **566**, 486-489 (2019).

4. Aryal, N. & Manousakis, E. Importance of electron correlations in understanding photoelectron spectroscopy and Weyl character of $MoTe_2$. *Phys. Rev. B* **99**, 035123 (2019).

5. Villaos, R. A. B. et al. Evolution of the electronic properties of ZrX_2 (X = S, Se, or Te) thin films under varying thickness. *J. Phys. Chem. C* **125**, 1134-1142 (2021).
